# Influence of Performance Status on the Effectiveness of Pembrolizumab Monotherapy in First-Line for Advanced Non-Small-Cell Lung Cancer: Results in a Real-World Population

**DOI:** 10.3390/biology10090890

**Published:** 2021-09-09

**Authors:** Rocío Jiménez Galán, Elena Prado-Mel, María Antonia Pérez-Moreno, Estefanía Caballano-Infantes, Sandra Flores Moreno

**Affiliations:** 1 Clinical Unit of Pharmacy, University Hospital Virgen del Rocio, Avenue Manuel Siurot, 41013 Seville, Spain; mariae.prado.sspa@juntadeandalucia.es (E.P.-M.); mantonia.perez.sspa@juntadeandalucia.es (M.A.P.-M.); estefania.caballano@juntadeandalucia.es (E.C.-I.); sandra.flores.sspa@juntadeandalucia.es (S.F.M.); 2 Andalusian Public Foundation for Health Research Management of Seville (FISEVI), 41013 Seville, Spain; 3 Instituto de Biomedicina de Sevilla (IBiS), 41013 Seville, Spain

**Keywords:** pembrolizumab, non-small-cell lung cancer, Eastern Cooperative Oncology Group Performance Status (ECOG PS)

## Abstract

**Simple Summary:**

Monotherapy with pembrolizumab is the standard of first-line treatment for patients with advanced non-small-cell lung cancer (NSCLC) and a programmed Death ligand-1 (PD-L1) tumor proportion score (TPS) ≥ 50%. However, pembrolizumab outcomes in real-world settings seem to be lower than those obtained in randomized clinical trials (RCTs). In this study, we analyzed a cohort of 88 patients with advanced NSCLC and PD-L1 TPS ≥ 50% treated in first-line with pembrolizumab and we investigated the influence of the Eastern Cooperative Oncology Group Stage Performance Status (ECOG PS) as a possible prognostic factor of survival. Our study showed that patients with PS ≥ 2 had poorer pembrolizumab outcomes with a significantly lower rate of response, progression-free survival (PFS) and overall survival (OS). ECOG PS was the only prognostic factor of PFS and OS identified. To confirm these results, patients with PS ≥ 2, should be studied in RCTs and include other tools to evaluate patients’ PS more objectively.

**Abstract:**

The KEYNOTE-024 clinical trial showed promising results for pembrolizumab in the first-line of treatment of advanced non-small-cell lung cancer (NSCLC). However, the profile of patients in real-world practice differs from those included in this clinical trial. Here, an observational single-center retrospective study was performed through a comparative analysis of clinical outcomes after pembrolizumab therapy according to the Eastern Cooperative Oncology Group Stage Performance Status (ECOG PS). Moreover, univariate and multivariate analyses were carried out to detect prognostic factors. In our cohort, 63.7% of patients had an ECOG PS of 0–1. Regarding response rate, 31.8% of patients had a partial response (PR), 19.3% had stable disease (SD) and 23.9% had progression disease. On the other hand, patients with ECOG PS ≥ 2 showed a significantly lower rate of PR and SD to pembrolizumab than patients with a PS of 0–1. The rate of response, median overall survival (OS) and progression-free survival (PFS) were significantly higher in patients with ECOG PS 0–1 than in those with ECOG PS ≥ 2. In the current study, we found ECOG PS as the only independent predictor of OS and PFS. Due to the ECOG PS scale being a subjective parameter, other tools are needed to identify treatment effectiveness to each patient.

## 1. Introduction

Immune checkpoint inhibitors (ICIs) play a key role in the treatment of non-small-cell lung cancer (NSCLC), showing successful results in patients with an advanced stage of this disease [1,2,3,4]. Currently, there are three ICIs, which have been approved by the Foods and Drug Administration (FDA) and the European Medicines Agency (EMA) for the treatment of stage IV NSCLC; atezolizumab [5] and pembrolizumab [6,7] have been approved for first-line treatment of metastatic NSCLC and atezolizumab and nivolumab for patients with locally advanced or metastatic NSCLC with progression after prior chemotherapy.

The role of immunotherapy has changed the approach to metastatic NSCLC [6,8,9]. The KEY-NOTE-024 clinical trial [6], conducted in patients with metastatic NSCLC and high PD-L1 expression levels (≥50%), showed the best results reported to date in terms of response rate, progression-free survival (PFS) and overall survival (OS). As a result, pembrolizumab has become the standard treatment of NSCLC for newly diagnosed patients with PD-L1 expression levels ≥ 50% and without EGFR (epidermal growth factor receptor) or ALK (anaplastic lymphoma kinase) positive mutations.

The strict inclusion criteria in clinical trials can sometimes make it difficult to extrapolate their results into actual clinical practice. In real life, approximately 30–40% [10] of patients with NSCLC have a PS of 2 at the time of diagnosis. However, this subgroup of patients was excluded from most of the randomized clinical trials (RCTs) evaluating ICIs in NSCLC [6,8,9]. Recently, results of a single-arm phase II trial [11] conducted in patients with NSCLC and a PS of 2 who received pembrolizumab as monotherapy showed worse outcomes compared with those obtained in the KEYNOTE-024 trial. Different results in terms of PFS, OS and response rate have been found across several real-world studies which enrolled 20–40% of PS 2 patients [12,13,14,15,16].

The aim of this retrospective study was to evaluate the influence of performance status on the clinical outcomes of pembrolizumab monotherapy as first-line treatment for patients with advanced or metastatic NSCLC in a real-world setting (effectiveness).

## 2. Materials and Methods

### 2.1. Study Design and Patients

We performed a single-center retrospective observational study of the use of pembrolizumab in a tertiary hospital of the Public Health System of Andalusia, Spain.

Inclusion criteria: all the patients who received pembrolizumab monotherapy (200 mg intravenous every three weeks) as first-line treatment for advanced NSCLC with a PD-L1 (Programmed Death-Ligand 1) tumor proportion score (TPS) ≥ 50% and negative mutations of EGFR/ALK between January 2017 and June 2020, with a follow-up cutoff established at 31 May 2021.

The study was designed in accordance with the Strengthening the Reporting of Observational Studies in Epidemiology (STROBE) reporting guidelines.

### 2.2. Study Variables and Analysis

The following variables were collected:

- Demographic data—patients’ age and sex.
- History of smoking.
- Diagnostic and treatment data—tumor histology, presence or absence of brain metastasis, disease stage and ECOG PS at the beginning of pembrolizumab treatment, PD-L1 TPS and reasons for the end of treatment.
- Effectiveness variables—response to the treatment considered as complete response (CR), partial response (PR), stable disease (SD), disease progression (DP) or unknown, overall survival (OS; months) and progression-free survival (PFS; months).

To perform the effectiveness analysis, we calculated the objective response rate to the treatment including CR and PR, OS (considered as the time between initiation of immunotherapy and date of death from any cause) and PFS (calculated as the time between the start of pembrolizumab and date of progression or death from any cause).

Additionally, we conducted a comparative analysis of treatment response (PR, SD and PD), according to ECOG PS at the beginning of treatment (PS of 0–1 vs. PS ≥ 2) and PD-L1 TPS (PD-L1 TPS ≥ 90% vs. < 90%). A univariate analysis was used to detect possible prognostic factors of OS and PFS (based on gender, age (<70 and ≥70 years), ECOG, tumor histology, PD-L1 TPS and the presence of brain metastasis), with the aim of performing a multivariate analysis.

The data and treatment information were obtained from the oncology pharmacy database and digital clinical records in the hospital.

### 2.3. Statistical Analysis

Continuous variables were expressed with measures of central tendency (mean or median) and measures of dispersion (standard deviation or inter-quartile range). Categorical variables were summarized with frequencies and percentages. The Mann–Whitney U test or Student’s *t*-test were used to compare continuous variables and Fischer’s exact test for categorical variables. Kolmogorov–Smirnov was used to test for the normality of data.

The probability of survival was estimated using the Kaplan–Meier method. Patients that did not experience the event (death and/or progression) before the end of the study were censored. We developed a multivariate analysis that identified the role of ECOG PS as a prognostic factor of OS and PFS. Univariate analysis was performed using the long-rank test to know the relationship between each of the variables and OS and PFS. Subsequently, the Cox proportional-hazards model was performed with those variables that had shown statistical significance in the univariate analysis. Hazard ratios and associated 95% confidence intervals were calculated with the Cox proportional-hazards model

The statistics program SPSS^®^ 24.0 was used and the threshold for statistical significance was established at a *p* < 0.05.

### 2.4. Ethics

In accordance with the Organic Protection of Data Law 15/1999, of 13 December (OPDL), and in order to protect patient’s confidential data, patients were identified by a corresponding numerical code. So, it was not necessary to obtain informed consent.

## 3. Results

### 3.1. Baseline Characteristics of the Study Population

A total of 88 patients were included in the study. Demographic and clinical characteristics are detailed in Table 1. The median age was 66 years (range of 46–85), most of the patients were males (78.4%) and were or had been smokers (90.9%). Predominant histology was adenocarcinoma, present in 68.2% of patients. A total of 4.5% of patients presented other histological subtypes (three with pleomorphic NSCLC and one patient with pulmonary sarcomatoid carcinoma). All patients had unresectable disease, most of them had stage IV disease (97.7%) and 15.9% had brain metastasis. A total of 63.7% of patients had an ECOG PS of 0–1 at the beginning of pembrolizumab treatment and the rest of patients had an ECOG ≥ 2, of which 7 patients had an ECOG of 3. A total of 29.5% of patients had a PD-L1 expression level ≥ 90% of tumor cells.

No statistically significant differences were found in baseline demographic and clinical characteristics among patients with an ECOG PS of 0–1 and an ECOG PS ≥ 2 (Table 2).

### 3.2. Pembrolizumab Outcomes in the Overall Population

All patients included in the study received at least one cycle of Pembrolizumab. The median number of cycles administered was six (range, 1–49).

Regarding the response to the treatment, no patients achieved complete response, 31.8% of patients had partial response (PR), 19.3% had stable disease (SD) and 23.9% of patients showed disease progression (Table 3).

Tumor response could not be assessed in 25.0% of patients due to death or clinical progression before first evaluation (censored).

**Figure 1:**
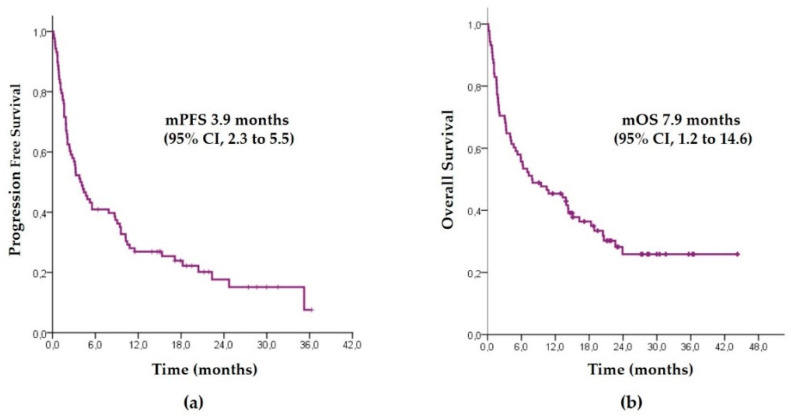
Estimation of probability of progression-free survival (**a**) and overall survival (**b**) through Kaplan–Meier curves in overall population. mPFS (median Progression Free Survival); mOS (median Overall Survival).

At the study cutoff date, with a median follow-up time of 23 months, 27 patients (30.7%) were alive and 71 patients (80.7%) had progressed. The median PFS (Figure 1a) was 3.9 months (95% CI, 2.3–5.6) and the estimated percentage of patients who did not have disease progression at 6 and 12 months was 27% and 24%, respectively. The median OS (Figure 1b) was 7.9 months (95% CI, 1.2–14.6) and the estimated percentage of patients who were alive at 6 and 12 months was 45% and 36%, respectively.

### 3.3. Results of Pembrolizumab according to ECOG PS

Table 4 includes the rates of partial response and stable disease according to ECOG PS (0–1 vs. ≥2). Patients with a PS ≥ 2 showed a significantly lower rate of partial response (5.7% vs. 26.1%; *p* = 0.014) than patients with a PS of 0–1. Based on PD-L1 expression levels, similar rates of PR and SD were found in patients with PD-L1 expression levels ≥ 90% and < 90% (34.6% vs. 30.6%, *p* = 0.71, and 19.2% and 19.4%, *p* = 0.99, respectively).

**Figure 2:**
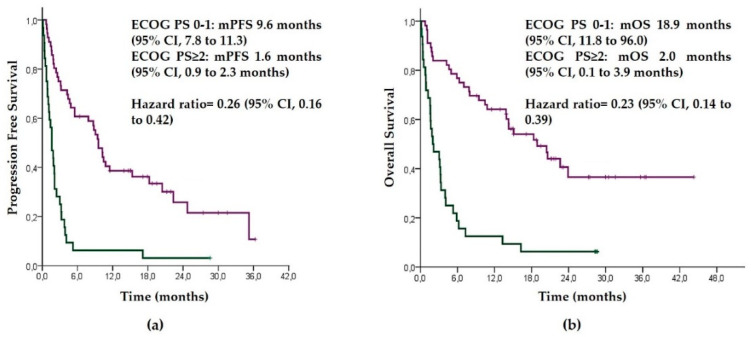
Comparative Kaplan–Meier curves of progression-free survival (**a**) and overall survival (**b**) according to an Eastern Cooperative Oncology Group Performance Status (ECOG PS) of 0–1 vs. ≥2. Line purple: ECOG PS 0-1; Line green: ECOG PS ≥2. mPFS (median Progression Free Survival); mOS (median Overall Survival).

Median PFS and OS (Figure 2) were significantly higher in patients with an ECOG PS of 0–1 compared with those with an ECOG PS ≥ 2 (18.9 vs. 2.0 months, *p* < 0.001, and 9.6 vs. 1.6 months, *p* < 0.001, respectively).

In the univariate analysis of PFS and OS (Table 5), the rest of the variables analyzed (sex, age (<70 vs. ≥70 years), histology (adenocarcinoma vs. non-adenocarcinoma), brain metastasis (yes vs. no) and PD-L1 TPS (≥90% vs. <90%)) did not show statistically significant differences according to ECOG PS. The median OS and PFS were higher in women than in men; however, this difference did not reach statistical significance. Significant histological differences were detected between women and men; 94.7% of women had adenocarcinoma NSCLC, compared with 60.9% of men (*p* = 0.005). In addition, no women had sarcomatoid or poorly differentiated NSCLC subtype. The rest of the baseline clinical characteristics were similar between both sexes.

Multivariate analysis adjusted by sex, age (<70 and ≥70 years), smoking habit, adenocarcinoma histology (yes vs. no) and presence of brain metastasis confirmed that the ECOG PS was the only independent predictor of PFS (HR = 0.25 (95% CI, 0.16–0.42); *p* < 0.001) and OS (HR = 0.23 (95% CI, 0.14–0.39); *p* < 0.001) in our cohort of patients.

## 4. Discussion

In this study, we present the clinical outcomes of pembrolizumab monotherapy as first-line treatment for patients with advanced or metastatic NSCLC in a real-world setting. The results obtained in our study are far from reaching those obtained in the KEYNOTE-024 study [6,17]. The median PFS (almost 6 months lower than expected) and OS, which did not reach 8 months, may raise questions about the benefit of immunotherapy in the treatment of NSCLC in real life. This aspect could be explained by the differences detected between patients included in our study and those included in the clinical trial. Baseline characteristics of patients enrolled in our study, related to sex, median age, patients with squamous histology and presence of brain metastasis, were comparable to those included in the pivotal clinical trial. However, while 34.6% of patients in our study had a PS ≥ 2, the KEYNOTE-024 study only included PS 0–1 patients. Despite the fact that approximately 34% of patients with NSCLC had a PS ≥ 2 at diagnosis [18], solid data on the benefit of ICIs in patients with an ECOG PS ≥ 2 are currently lacking because these patients had been excluded from RCTs. To date, we have only the results of the phase 2 clinical trial PePS2 [11] that evaluated the benefit of pembrolizumab in a selected population of 60 patients with advanced NSCLC and a PS ≥ 2. Nevertheless, the study population was heterogeneous, including patients treated with pembrolizumab in first and subsequent lines of therapy and with PD-L1 expression levels <50% and ≥50%. Only 40% of the patients included in this study received pembrolizumab as first-line treatment, while the remaining patients receive it in later lines. Besides this and unlike our study, there were patients treated with pembrolizumab in first-line treatment with a PD-L1 TPS < 50%, which makes the comparability with our results difficult. According to our findings, the median OS and PFS in patients treated with pembrolizumab in a first-line setting were shorter than in the KEYNOTE-024 and KEYNOTE-042 clinical trials [6,9] (7.9 and 4.3 months, respectively), but even so, the results of these studies were better than ours. With respect to the results based on PD-L1 expression levels, regardless of the line of therapy received, median OS and PFS were 14.6 and 12.6 months, respectively. Baseline characteristics of patients were similar to ours, although the proportion of men in our study was higher (55% vs. 75%).

Another retrospective study, focused on PS 2 patients treated with pembrolizumab in a first-line setting, showed results consistent with ours, with median PFS and OS of 2.4 and 3 months, respectively [19]. On the other hand, based on progression-free rate (PFR) at six months after beginning of treatment, authors categorized pembrolizumab outcomes according to three different scenarios: (i) optimistic scenario (6-month PFR of 60%), corresponding to the outcome observed in patients with an ECOG PS of 0–1 in the KEYNOTE-024 randomized trial; (ii) intermediate scenario (6-month PFR of 45%), corresponding to the outcome observed in patients with ECOG PS 2 treated with carboplatin plus pemetrexed; (iii) pessimistic scenario (6-month PFR of 30%), corresponding to the outcome observed in patients with ECOG PS 2 treated with single-agent pemetrexed. In this study, the 6-month PFR was 27%, corresponding with the most pessimistic scenario. However, these results should not be extrapolated to all patients with a PS of 2 because some patients obtain lasting responses and even improve PS after treatment [12,19]. In addition, in this study, patients with a PS of 2 due to comorbidities had a significantly better prognosis than patients whose poor PS was determined by the disease burden.

The retrospective study carried out by Sehgal et al. [20] included 74 patients with advanced NSCL treated in monotherapy with pembrolizumab. The purpose of this study was to determine the influence of PS on PFS and OS. The proportion of patients with PS ≥ 2 was similar to our study (39.2%) and they identified this variable as an independent predictor of PFS and OS. Median PFS and OS in PS ≥ 2 patients, compared with PS 0–1 patients, were 2.3 vs. 7.9 months and 4.1 vs. 23.2 months, respectively. As in the PePS2 clinical trial [11], this study included patients treated with pembrolizumab beyond the first line (27.0%) and with a PD-L1 expression level < 50% and ≥ 50%.

Friedlaender et al. [21] found similar results in a population of 302 patients with advanced NSCLC and PD-L1 expression ≥ 50% treated with pembrolizumab, but including first and subsequent lines of treatment. Median OS in PS ≤ 1 patients was not reached, probably due to an insufficient follow-up period (8.6 months).

Additionally, we have found some studies focused on patients with PD-L1 ≥ 50% treated in monotherapy with pembrolizumab in first-line setting, such as those in our work. The study published by Frost et al. [10] showed a significantly lower PFS and OS in PS ≥ 2 patients (2.2 and 6.6 months, respectively) with a proportion of patients with a PS ≥ 2 of 24.8%. As a limitation of this study, it should be mentioned that the median age and proportion of patients with squamous histology were significantly higher in patients with a PS ≥ 2 than in patients with a PS of 0–1, which could have influenced the results. In contrast, in our cohort, there were no statistically significant differences between these two subgroups of patients. In the study conducted by Alessi et al. [20], a low proportion of PS = 2 patients were included (16.7%), but also median PFS and OS were shorter in patients with PS = 2 (4 and 7.4 months, respectively). In this study, baseline characteristics were balanced according to PS.

Regarding the response rate, inconsistent results have been obtained across different studies. In our study, no patients had a complete response and the partial response rate (PRR) in the overall population was slightly lower than in the KEYNOTE-024; we also found a significant decrease in PRR in patients with a PS ≥ 2. In other studies, the objective response rate (partial plus complete response) in PS ≥ 2 patients ranged between 17.9% and 45% [12,20,21,22,23]. In contrast with our results, most authors have not found significant differences according to PS [12,20,23], with the exception of Alessi et al. [22], who detected a significantly poorer objective response rate in patients with a PS of 2 (43.1 vs. 25.6%), although this variable in our cohort with a PS ≥ 2 was notably lower.

Therefore, despite the heterogeneous population included in the different studies, available evidence suggests that PS is a determinant factor in the benefit of pembrolizumab in patients with advanced NSCLC. Pembrolizumab has been evaluated in randomized clinical trials in patients with a PS of 0–1 exclusively. However, regulatory agencies have approved pembrolizumab independently of PS. Therefore, most clinical practice guidelines recommend pembrolizumab in first-line settings in patients with PD-L1 ≥ 50% without considering ECOG PS. The latest ESMO guideline limits the use of pembrolizumab in first line to an ECOG PS of 0–1 with a level IA recommendation [24]. For PS ≥ 2, carboplatin-based or single-agent chemotherapy are recommended, independently of PD-L1 expression and ICIs are mentioned as an option in this subgroup of patients, but with a level IIIB recommendation, due to lack of data/evidence.

ECOG PS is a subjective parameter that categorizes patients with very different characteristics. Each level of the scale can include a very heterogeneous patient population. Two physicians could categorize the same patient with a different PS or patients-reported PS could differ from physician-reported PS. This scale does not consider aspects such as age, tumor burden, comorbidities, fragility or polypharmacy separately. Because of the high interindividual variability inherent to the ECOG scale [10], it is essential, when establishing criteria for access to treatments, to incorporate other parameters that provide a better approach to the analysis of the benefit of these therapies. Therefore, it should be necessary to incorporate, together with the ECOG, other tools, such as a frailty index that assesses the patient’s intrinsic comorbidities, and it would be necessary to design studies aimed to correctly define this subpopulation and establish the characteristics of the patients who could clearly benefit from this therapy.

As a limitation of this study, its single-center character should be noted, which made the number of patients who have received treatment limited. However, this number is in accordance with other studies or even higher. We could also mention the inherent limitations in the retrospective design of the study, with the potential risk of bias that this may entail. We included a low proportion of patients with other histologies, which have been related to worse prognostic. This could explain the worse results of pembrolizumab in the overall population compared with other studies. Nevertheless, non-significant differences were found in histologic subtypes according to ECOG and the univariate/multivariate analyses did not identify this aspect as a prognostic factor in our patients.

Despite these limitations, our results are consistent with other retrospective studies, in which there seems to be an association between performance status and response to treatment. This denotes the importance of identifying which profile of patients could best benefit from the treatment with pembrolizumab. In contrast with other published studies, we included a selected population and balanced characteristics according to ECOG that allowed predicting the effect of PS.

## 5. Conclusions

In conclusion, outcomes of pembrolizumab treatment for advanced NSCLC in a first-line setting in our study were considerably worse than those in the KEYNOTE-024 and other real-world life studies. PS was the single independent predictor of OS and PFS recognized in our study and, in the same way, patients with a PS ≥ 2 also had a significantly lower response to pembrolizumab. To determine the real benefit of ICIs in patients with a PS ≥ 2, this subgroup of patients should be studied in RCTs. Because the ECOG PS scale is a subjective parameter, other tools are needed to analyze the PS of patients more precisely and to provide optimal treatment to each patient.

## Figures and Tables

**Table 1:** Baseline demographic and clinical characteristics of patients.

Characteristics	Values, n (%)
Sex	
Male	69 (78.4)
Female	19 (21.6)
**Age median (range), y**	67 (46–85)
**Smoking history**	
Yes	80 (90.9)
No	8 (9.1)
**Histology**	
Adenocarcinoma	60 (68.2)
Squamous	18 (20.5)
NSCLC poorly differentiated	6 (6.8)
Others	4 (4.5)
**Disease stage**	
IIIB	2 (2.3)
IV	86 (97.7)
**Brain metastases**	
Yes	14 (15.9)
No	74 (84.1)
**ECOG PS**	
0	18 (20.5)
1	38 (43,2)
2	25 (28.4)
3	7 (8.0)
**PD-L1 TPS%**	
<90%	62 (70.5)
≥90%	26 (29.5)

NSCLC, non-small-cell lung cancer; ECOG PS, Eastern Cooperative Oncology Group performance status; PD-L1, programmed death ligand 1; TPS, tumor proportion score.

**Table 2:** Baseline demographic and clinical characteristics of patients according to ECOG PS.

Characteristics	ECOG 0–1 n (%)	ECOG ≥ 2 n (%)	*p*-Value
Sex			0.304
Male	42 (75)	27 (84.4)	
Female	14 (25)	5 (15.6)	
**Age median (range), y**	66.8 (46–84)	66.1 (49–85)	0.68
**Smoking history**	50 (89.3)	30 (93.8)	0.483
**Histology**			0.331
Adenocarcinoma	41(73.2)	19 (54.4)	
Squamous	11 (19.6)	7 (21.9)	
NSCLC poorly differentiated	2 (3.6)	4 (12.5)	
Others	2 (3.6)	2 (6.3)	
**Disease stage**			0.582
**IIIB**	1 (1.8)	1 (3.1)	
**IV**	55 (98.2)	31 (96.9)	
**Brain metastases**			0.607
Yes	8 (14.3)	6 (18.8)	
No	48 (85.7)	26 (81.3)	
**PD-L1 TPS%**			0.453
<90%	41(73.2)	21 (65.6)	
≥90%	15 (26.8)	11 (34.4)	

ECOG PS, Eastern Cooperative Oncology Group performance status; PD-L1, Programmed Death-Ligand 1; TPS, tumor proportion score.

**Table 3:** Treatment outcomes in the overall population.

Response	N (%)
CR	-
PR	28 (31.8)
SD	17 (19.3)
PD	21 (23.9)
NE	22 (25.0)

CR, complete response; PR, partial response; SD, stable disease; PD, progressive disease; NE, not evaluated.

**Table 4:** Treatment outcomes according to ECOG PS.

Response	ECOG PS 0–1	ECOG PS ≥ 2
PR, n (%)	23 (26.1)	5 (5.7)
SD, n (%)	16 (18.2)	1 (1.1)
PD, n (%)	11 (12.5)	10 (11.4)

PR, partial response; SD, stable disease; PD, progressive disease.

**Table 5:** Univariate analysis of progression-free survival and overall survival.

	Progression Free Survival			Overall Survival		
Variable	Median (95% CI)	HR (95% CI)	*p*-Value	Median (95% CI)	HR (95% CI)	*p*-Value
**Sex**		0.75 (0.42–1.34)	0.33		0.64 (0.33–1.23)	0.175
Female	9.06 (1.72–4.98)			18.9 (4.4–33.9)		
male	3.2 (1.41–4.98)			7.03 (4.56–9.47)		
**Age**		0.96 (0.59–1.56)	0.86		0.98 (0.59–1.65)	0.965
<70	3.76 (1.33–6.21)			7.9 (0–17.3)		
≥70	3.9 (0.71–7.09)			7.03 (1.94–12.13)		
**Smoking history**		0.775 (0.33–1.79)	0.538		0.69 (0.27–1.71)	0.42
Yes	3.7 (2.08–5.44)			7.03 (2.03–12.04)		
No	4.4 (0–14.7)			10.8 (0–28.3)		
**ECOG PS**		0.25 (0.16–0.42)	<0.001		0.23 (0.13–0.38)	<0.001
0–1	9.57 (7.82–11.32)			18.9 (11.77–26.03)		
ECOG ≥ 2	1.63 (0.99–2.28)			2.0 (0.11–3.89)		
**Histology**		0.79 (0.48–1.31)	0.36		0.73 (0.43–1.23)	0.238
Adenocarcinoma	4.4 (2.48–6.32)			9.47 (0.19–18.74)		
Non-adenocarcinoma	2.1 (0.46–3.74)			5.9 (0.67–11.3)		
**PD-L1 TPS**		0.77 (0.46– 1.28)	0.32		0.75 (0.44–1.28)	0.283
<90%	4.1 (2.29–5.9)			9.47 (1.26–17.67)		
≥90%	3.27 (0–7.8)			5.3 (0–11.17)		
**Brain Metastasis**		0.70 (0.38–1.29)	0.25		0.62 (0.33–1.17)	0.139
No	4.23 (1.98–6.48)			10.47 (3.52–17.42)		
Yes	3.2 (1.12–5.28)			4.1 (2.33–5.87)		

HR, hazard ratio; ECOG PS, Eastern Cooperative Oncology Group performance status; PD-L1, programmed cell death ligand 1; TPS, tumor proportion score.

## Data Availability

The data presented in this study are not publicly available.

## References

[1] Borghaei H., Paz-Ares L., Horn L., Spigel D.R., Steins M., Ready N.E., Chow L.Q., Vokes E.E., Felip E., Holgado E. (2015). Nivolumab versus Docetaxel in Advanced Nonsquamous Non-Small-Cell Lung Cancer. N. Engl. J. Med..

[2] Herbst R.S., Baas P., Kim D.-W., Felip E., Pérez-Gracia J.L., Han J.-Y., Molina J., Kim J.-H., Arvis C.D., Ahn M.-J. (2016). Pembrolizumab versus Docetaxel for Previously Treated, PD-L1-Positive, Advanced Non-Small-Cell Lung Cancer (KEYNOTE-010): A Randomised Controlled Trial. Lancet Lond. Engl..

[3] Brahmer J., Reckamp K.L., Baas P., Crinò L., Eberhardt W.E.E., Poddubskaya E., Antonia S., Pluzanski A., Vokes E.E., Holgado E. (2015). Nivolumab versus Docetaxel in Advanced Squamous-Cell Non-Small-Cell Lung Cancer. N. Engl. J. Med..

[4] Rittmeyer A., Barlesi F., Waterkamp D., Park K., Ciardiello F., von Pawel J., Gadgeel S.M., Hida T., Kowalski D.M., Dols M.C. (2017). Atezolizumab versus Docetaxel in Patients with Previously Treated Non-Small-Cell Lung Cancer (OAK): A Phase 3, Open-Label, Multicentre Randomised Controlled Trial. Lancet Lond. Engl..

[5] Socinski M.A., Jotte R.M., Cappuzzo F., Orlandi F., Stroyakovskiy D., Nogami N., Rodríguez-Abreu D., Moro-Sibilot D., Thomas C.A., Barlesi F. (2018). Atezolizumab for First-Line Treatment of Metastatic Nonsquamous NSCLC. N. Engl. J. Med..

[6] Reck M., Rodríguez-Abreu D., Robinson A.G., Hui R., Csőszi T., Fülöp A., Gottfried M., Peled N., Tafreshi A., Cuffe S. (2016). Pembrolizumab versus Chemotherapy for PD-L1-Positive Non-Small-Cell Lung Cancer. N. Engl. J. Med..

[7] Gandhi L., Rodríguez-Abreu D., Gadgeel S., Esteban E., Felip E., De Angelis F., Domine M., Clingan P., Hochmair M.J., Powell S.F. (2018). Pembrolizumab plus Chemotherapy in Metastatic Non-Small-Cell Lung Cancer. N. Engl. J. Med..

[8] Boyer M., Şendur M.A.N., Rodríguez-Abreu D., Park K., Lee D.H., Çiçin I., Yumuk P.F., Orlandi F.J., Leal T.A., Molinier O. (2021). Pembrolizumab Plus Ipilimumab or Placebo for Metastatic Non-Small-Cell Lung Cancer With PD-L1 Tumor Proportion Score ≥50%: Randomized, Double-Blind Phase III KEYNOTE-598 Study. J. Clin. Oncol. Off. J. Am. Soc. Clin. Oncol..

[9] Mok T.S.K., Wu Y.-L., Kudaba I., Kowalski D.M., Cho B.C., Turna H.Z., Castro G., Srimuninnimit V., Laktionov K.K., Bondarenko I. (2019). Pembrolizumab versus Chemotherapy for Previously Untreated, PD-L1-Expressing, Locally Advanced or Metastatic Non-Small-Cell Lung Cancer (KEYNOTE-042): A Randomised, Open-Label, Controlled, Phase 3 Trial. Lancet Lond. Engl..

[10] Passaro A., Spitaleri G., Gyawali B., de Marinis F. (2019). Immunotherapy in Non-Small-Cell Lung Cancer Patients With Performance Status 2: Clinical Decision Making With Scant Evidence. J. Clin. Oncol. Off. J. Am. Soc. Clin. Oncol..

[11] Middleton G., Brock K., Savage J., Mant R., Summers Y., Connibear J., Shah R., Ottensmeier C., Shaw P., Lee S.-M. (2020). Pembrolizumab in Patients with Non-Small-Cell Lung Cancer of Performance Status 2 (PePS2): A Single Arm, Phase 2 Trial. Lancet Respir. Med..

[12] Frost N., Kollmeier J., Misch D., Vollbrecht C., Grah C., Matthes B., Pultermann D., Olive E., Raspe M., Ochsenreither S. (2021). Pembrolizumab as First-Line Palliative Therapy in PD-L1 Overexpressing (≥50%) NSCLC: Real-World Results with Special Focus on PS ≥ 2, Brain Metastases, and Steroids. Clin. Lung Cancer.

[13] Tambo Y., Sone T., Shibata K., Nishi K., Shirasaki H., Yoneda T., Araya T., Kase K., Nishikawa S., Kimura H. (2020). Real-World Efficacy of First-Line Pembrolizumab in Patients With Advanced or Recurrent Non-Small-Cell Lung Cancer and High PD-L1 Tumor Expression. Clin. Lung Cancer.

[14] Cavaille F., Peretti M., Garcia M.E., Giorgi R., Ausias N., Vanelle P., Barlesi F., Montana M. (2021). Real-World Efficacy and Safety of Pembrolizumab in Patients with Non-Small Cell Lung Cancer: A Retrospective Observational Study. Tumori.

[15] Amrane K., Geier M., Corre R., Léna H., Léveiller G., Gadby F., Lamy R., Bizec J.-L., Goarant E., Robinet G. (2020). First-Line Pembrolizumab for Non-Small Cell Lung Cancer Patients with PD-L1 ≥50% in a Multicenter Real-Life Cohort: The PEMBREIZH Study. Cancer Med..

[16] Velcheti V., Chandwani S., Chen X., Pietanza M.C., Burke T. (2019). First-Line Pembrolizumab Monotherapy for Metastatic PD-L1-Positive NSCLC: Real-World Analysis of Time on Treatment. Immunotherapy.

[17] Reck M., Rodríguez-Abreu D., Robinson A.G., Hui R., Csőszi T., Fülöp A., Gottfried M., Peled N., Tafreshi A., Cuffe S. (2019). Updated Analysis of KEYNOTE-024: Pembrolizumab Versus Platinum-Based Chemotherapy for Advanced Non-Small-Cell Lung Cancer With PD-L1 Tumor Proportion Score of 50% or Greater. J. Clin. Oncol. Off. J. Am. Soc. Clin. Oncol..

[18] Lilenbaum R.C., Cashy J., Hensing T.A., Young S., Cella D. (2008). Prevalence of Poor Performance Status in Lung Cancer Patients: Implications for Research. J. Thorac. Oncol. Off. Publ. Int. Assoc. Study Lung Cancer.

[19] Facchinetti F., Mazzaschi G., Barbieri F., Passiglia F., Mazzoni F., Berardi R., Proto C., Cecere F.L., Pilotto S., Scotti V. (2020). First-Line Pembrolizumab in Advanced Non-Small Cell Lung Cancer Patients with Poor Performance Status. Eur. J. Cancer Oxf. Engl..

[20] Sehgal K., Gill R.R., Widick P., Bindal P., McDonald D.C., Shea M., Rangachari D., Costa D.B. (2021). Association of Performance Status With Survival in Patients With Advanced Non-Small Cell Lung Cancer Treated With Pembrolizumab Monotherapy. JAMA Netw. Open.

[21] Friedlaender A., Metro G., Signorelli D., Gili A., Economopoulou P., Roila F., Banna G., De Toma A., Camerini A., Christopoulou A. (2020). Impact of Performance Status on Non-Small-Cell Lung Cancer Patients with a PD-L1 Tumour Proportion Score ≥50% Treated with Front-Line Pembrolizumab. Acta Oncol. Stockh. Swed..

[22] Alessi J.V., Ricciuti B., Jiménez-Aguilar E., Hong F., Wei Z., Nishino M., Plodkowski A.J., Sawan P., Luo J., Rizvi H. (2020). Outcomes to First-Line Pembrolizumab in Patients with PD-L1-High (≥50%) Non-Small Cell Lung Cancer and a Poor Performance Status. J. Immunother. Cancer.

[23] Matsubara T., Seto T., Takamori S., Fujishita T., Toyozawa R., Ito K., Yamaguchi M., Okamoto T. (2021). Anti-PD-1 Monotherapy for Advanced NSCLC Patients with Older Age or Those with Poor Performance Status. OncoTargets Ther..

[24] Planchard D., Popat S., Kerr K., Novello S., Smit E.F., Faivre-Finn C., Mok T.S., Reck M., Van Schil P.E., Hellmann M.D. (2018). Metastatic Non-Small Cell Lung Cancer: ESMO Clinical Practice Guidelines for Diagnosis, Treatment and Follow-Up. Ann. Oncol. Off. J. Eur. Soc. Med. Oncol..

